# From Preparation to Bioactivity: A Comparative Study on Preparation Methods and Characterization of Postbiotics

**DOI:** 10.1002/fsn3.70294

**Published:** 2025-05-19

**Authors:** Amtalsaboor Almahbashi, Evrim Gunes Altuntas

**Affiliations:** ^1^ Ankara University Biotechnology Institute Ankara Turkey

**Keywords:** antioxidant activity, lactic acid bacteria, phenolic and flavonoid compounds, postbiotic preparation methods, total phenolic content

## Abstract

Postbiotics are bioactive compounds derived from bacterial metabolism that offer various health benefits in food applications. This study aimed to compare three methods for preparing postbiotics: cell‐free supernatant (CFS), thermal treatment, and enzymatic treatment with ultrasonication by using lactic acid bacteria (LAB): *Lactiplantibacillus plantarum* F2, *Leuconostoc mesenteroides* AF1, and *Pediococcus pentosaceus* 50. The focus was on assessing cell viability and antimicrobial activity, followed by characterizing the CFS through additional analyses of antioxidant activity, total phenolic content (TPC), and the presence of phenolic and flavonoid compounds. The results demonstrated that both the CFS and thermal treatment methods effectively removed viable bacterial cells, whereas enzymatic treatment combined with ultrasonication exhibited lower efficacy. Antimicrobial activity varied based on both the bacterial strain and the preparation method. Specifically, *L. plantarum* F2 and *L. mesenteroides* AF1 exhibited higher antimicrobial activity when using the CFS method, whereas *P. pentosaceus* 50 showed greater efficacy after complete thermal treatment. Additionally, *L. plantarum* F2 demonstrated the highest antioxidant capacity and TPC, measuring 76.11 ± 6.21 mg/L and 125 ± 6.6 mg GAE/mL, respectively. This was correlated with elevated lactic acid production (17.2 ± 0.1 mg/mL) and acetic acid production (6.25 ± 0.07 mg/mL). *P. pentosaceus* 50 had the highest concentration of citric acid (1.47 ± 0.04 mg/mL), while *L. mesenteroides* AF1 recorded the highest level of succinic acid at 1.10 ± 0.02 mg/mL. These findings highlight the impact of preparation methods and strain‐specific characteristics on the bioactivity of postbiotics, providing valuable insights for optimizing postbiotic production in food preservation applications.

## Graphical abstract

Postbiotics, bioactive compounds from bacterial metabolism, offer health benefits in food applications. This study compares three preparation methods: cell‐free supernatant (CFS), thermal treatment, and enzymatic treatment with sonication—using Lactiplantibacillus plantarum F2, *Leuconostoc mesenteroides* AF1, and *Pediococcus pentosaceus* 50.

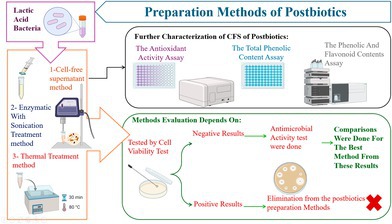

**Abbreviations**
CFS: cell‐free supernatant
CHT: complete heating treatment
DPPH: 2,2‐diphenyl‐1‐picrylhydrazyl
LAB: lactic acid bacteria
MHT: mixed heating treatment
MRM: multiple reaction monitoring
MRS: deMan, Rogosa, and Sharpe
Na_2_CO_3_
: sodium carbonate
TPC: total phenolic content
TSA: tryptic soy agar
TSB: tryptic soy broth

## 1 Introduction

Postbiotics have gained increasing importance in recent decades due to their bioactive compounds, which are effectively used in food preservation applications. According to the International Scientific Association for Probiotics and Prebiotics (ISAPP), postbiotics are “preparations of inanimate microorganisms and/or their components that confer a health benefit on the host” (Salminen et al. 2021). This definition encompasses inactivated cells, their particles, secreted compounds in CFSs, or combinations thereof (Sharafi et al. 2023). Lactic acid bacteria (LAB) are considered one of the most important sources for postbiotic production due to their ability to produce diverse bioactive molecules, such as organic acids, bacteriocins, enzymes, peptides, and polysaccharides, which exhibit antimicrobial, antioxidant, and anti‐inflammatory properties (Moradi et al. 2020). Various LAB species, including *Lactobacillus*, *Bifidobacterium*, and *Streptococcus*, are commonly used for postbiotic production, each yielding distinct bioactive profiles (Celik et al. 2024). The selection of these bacteria depends on the bioactive compounds produced through different metabolic pathways. For example, *Lactobacillus plantarum* primarily utilizes homofermentative or heterofermentative pathways, producing lactic acid, plantaricins, and other antimicrobial peptides (Brizuela et al. 2019). *Bifidobacterium bifidum* follows the bifid shunt pathway, generating exopolysaccharides and short‐chain fatty acids that contribute to its health benefits (Chen et al. 2021). *Streptococcus thermophilus* , on the other hand, is known for its proteolytic pathways, resulting in bioactive peptides and other functional metabolites (Li et al. 2022). The distinction between these metabolic pathways results in varying functional properties of the postbiotics they produce.

When comparing probiotics and postbiotics, postbiotics have notable advantages, especially in food preservation. Probiotics rely on live microorganisms for health benefits, making them vulnerable to factors like heat and stomach acids, which can diminish their efficacy (Echresh et al. 2024; Rahmati‐Joneidabad et al. 2024). They may also pose risks such as adverse reactions and concerns about antibiotic resistance (Zavišić et al. 2023). In contrast, postbiotics do not require viability, making them more stable and enhancing their shelf life (Pimentel et al. 2023). They eliminate risks associated with live bacteria, such as pathogenicity, making them a safer and more reliable option for food preservation (Zhong et al. 2024). These advantages position postbiotics as a preferable and more practical option in food preservation.

Postbiotics have gained significant attention in food preservation due to their antimicrobial properties, which help reduce food‐borne microbes and extend shelf life (İçier et al. 2022). Various studies have explored their application across different food products, showcasing their effectiveness against diverse pathogens. In poultry products, postbiotics from *Pediococcus acidilactici* at a concentration of 10% reduced *Salmonella typhimurium* levels by 2.1 log on chicken drumsticks. Additionally, combining *P. acidilactici* with 1% lactic acid lowered *Listeria monocytogenes* by 1.1 log (*p* < 0.05) and minimized mesophilic aerobic bacteria counts without altering the drumsticks' color (*p* > 0.05), effectively extending shelf life (İncili et al. 2022). In dairy products, *L. plantarum* S62 postbiotics at concentrations of 0.45% (v/v) and 0.5% (v/v) exhibited the highest decontamination effects against milk contaminated with *Listeria innocua* and *Escherichia coli* , respectively (Hasnaoui et al. 2023). Similarly, in beverages, *P. pentosaceus* IO1 postbiotics at 10% (v/v) reduced *S. aureus* by 1.42 log CFU/mL in pasteurized orange juice and by 1.03 log CFU/mL in unpasteurized orange juice (Adesina and Oluwafemi 2022). Postbiotics have also shown promise in preserving fresh produce. Dipping apples, grapes, and bananas in postbiotics from *L. plantarum* DMR14 extended their shelf life compared to untreated control fruits (Islam et al. 2023). In bakery products, the direct application of *L. plantarum* 4F postbiotics on bread effectively inhibited *Aspergillus fumigatus* growth, keeping treated samples intact after 6 weeks of incubation. In contrast, about 40% of the control sample's surface was covered by *A. fumigatus* after just 3 days (El Oirdi et al. 2021). These findings highlight the versatility and potential of postbiotics as natural preservatives in various food products, making them a promising alternative for enhancing food safety and extending shelf life.

Several methods have been developed for preparing postbiotics, each offering distinct advantages and challenges (Rafique et al. 2023). These methods aim to extract bioactive compounds from microbial cells, including heat treatment, ultrasonication, high‐pressure processing, filtration techniques, and enzymatic hydrolysis (Moradi et al. 2020). The advantages of these techniques include their ability to effectively inactivate microorganisms, preserve bioactive compounds, enhance the release of intracellular components, and provide high purity, with enzymatic hydrolysis offering the benefit of controlled breakdown of microbial cell walls (Suthar et al. 2025). However, these methods also present certain challenges, such as the potential degradation of heat‐sensitive compounds, the requirement for specialized equipment and precise control, high operational costs, energy intensity, loss of smaller molecules, and the complexity of enzyme selection and optimizing hydrolysis conditions. Given the differences in these methods, the choice of postbiotic preparation technique must be tailored to the specific bacterial strain used, as each bacterium may respond differently based on the bioactive compounds it produces. Therefore, selecting the optimal method for postbiotic preparation is highly dependent on both the bacterial species and the desired bioactive compounds. This underscores the necessity of comparing postbiotic preparation methods across different bacterial strains to ensure that the most effective technique is chosen for each strain, leading to the highest yield and functionality of bioactive compounds (Rafique et al. 2023). The goal of this research is to compare three methods for preparing postbiotics from three distinct LAB strains, considering cell viability and antimicrobial properties, and evaluating their effectiveness for food preservation applications. Additionally, the study will assess the antioxidant activity, total phenolic content (TPC), and profiles of phenolic and flavonoid compounds in the CFS of these bacteria.

## 2 Experimental Section

### 2.1 Bacterial Strains and Growth Conditions

*Lactiplantibacillus plantarum* F2, *Pediococcus pentosaceus* 50, and *Leuconostoc mesenteroides* AF1 were used for postbiotic production (Çelik (2022), TÜBİTAK Project No. 119O343). The bacteria were cultured in de Man, Rogosa, and Sharpe Broth (MRS) (Condalab, Spain) with a 1% inoculum and incubated at 35°C for 18–24 h.

*Escherichia coli* ATCC 25922, *Staphylococcus aureus* ATCC 43300, *Listeria monocytogenes* ATCC 7644, and *Salmonella Enteritidis* ATCC 13076 (Ankara University Biotechnology Institute bacterial culture collection, Ankara‐Turkey) served as indicator microorganisms in antimicrobial assays. Indicator bacteria were activated in Tryptic Soy Broth (TSB) (Merck, Germany) at 37°C for 18–24 h, except *L. monocytogenes*, which was incubated at 30°C.

### 2.2 Preparation of Postbiotics

Three distinct methods were employed to obtain postbiotics from three LAB isolates: (1) cell‐free supernatant (CFS) preparation, (2) enzymatic lysis combined with ultrasonication, and (3) heat treatment. To facilitate a more comprehensive comparison and better understand the sources of antimicrobial activity, subgroups were established within methods 2 and 3. These subgroups included: (a) treated cells alone, subjected to either heat or enzymatic lysis with ultrasonication; (b) treated cells combined with untreated CFS, aimed at minimizing potential adverse effects of heat or enzymatic‐ultrasonication treatments on the bioactive compounds and metabolites in the CFS; and (c) complete preparations, where treated cells and their metabolites underwent either heat or enzymatic‐ultrasonication treatments together. This approach enabled the evaluation of each component's contribution to antimicrobial activity while assessing whether the treatments could enhance the beneficial properties of the resulting postbiotics.

#### 2.2.1 CFS Method

The supernatant was prepared by modifying the method described by Altuntaş et al. (2010). Each bacterial isolate was activated in MRS Broth at 35°C for 24 h, centrifuged at 4000 × *g* for 10 min at 4°C (Beckman Coulter Allegra X‐15R Centrifuge, USA), and the supernatant was collected. It was then filtered through a 0.45 μm membrane filter (Microcult, Haimen, China) to remove any remaining cells.

#### 2.2.2 Enzymatic Lysis With Ultrasonication Method

The enzymatic treatment involved three subgroups. The first subgroup focused on cell lysis, following the methodology described by Aguilar‐Toalá et al. (2019). In summary, after activating the bacteria and incubating for 24 h, a centrifugation step was conducted at 4000 × *g* for 10 min at 4°C. The cells were washed twice with PBS (Sigma‐Aldrich, USA) and then resuspended in 10 mL of PBS. Lysozyme (Applichem, Darmstadt, Germany) was added at a concentration of 1 mg/mL, and the resulting suspension was incubated at 37°C for 150 min. Subsequently, the cells underwent five 1‐min bursts of ultrasonication at 60 Hz (Sonics Vibra Cell, Newtown, USA), with a 1‐min rest in an ice bath maintained at 10°C following each burst. The second subgroup was a mixed enzyme‐treated sample, which was created by mixing the cell lysis solution from the first subgroup with the CFS prepared in the initial group of postbiotics at a 1:1 ratio. The third subgroup was a complete enzyme‐treated sample that involved adding lysozyme at a concentration of 1 mg/mL to the 24‐h incubated bacteria in MRS broth, followed by the same incubation and ultrasonication process as in the first subgroup. All subgroups were stored at 4°C in the dark until further use.

#### 2.2.3 Heat Treatment Method

Heat treatment is a widely used method for preparing effective postbiotics, as it can inactivate cells while maintaining their antimicrobial properties. Various temperatures can be applied, but a lower temperature is preferred to preserve antimicrobial activity and ensure the cells are nonviable. In this study, 80°C was selected based on a preliminary test conducted in our lab. The method was adapted from Ou et al. (2011) with slight modifications. Briefly, three experimental subgroups were prepared: (1) Heated cells (HC) were prepared by centrifuging activated bacterial cultures, washing the pellet with (PBS), resuspending the cells in PBS, and subjecting them to heat treatment in a water bath at 80°C for 30 min (MIPR, Ankara, Türkiye); (2) The complete heat‐treated (CHT) subgroup, consisting of heated cells with their metabolites, was prepared by heat‐treating activated bacterial cultures in MRS broth in a water bath at 80°C for 30 min; (3) The mixed heat‐treated (MHT) subgroup, consisting of heat‐treated cells combined with untreated supernatant, was prepared by mixing equal volumes (1:1 ratio) of heat‐treated cells (as described in the HC subgroup) and CFS obtained without heat treatment, as outlined in the CFS postbiotic preparation method. These heat‐treated postbiotic subgroups were stored at 4°C for immediate use or at −20°C for long‐term storage.

### 2.3 Cell Viability Test

To confirm that the postbiotics prepared by different methods were free of viable cells, a cell viability test was conducted according to Altuntas et al. (2010). Briefly, each sample (100 μL) was spread on sterilized MRS agar (Condalab, Madrid, Spain) and incubated overnight at 35°C to detect any remaining viable cells. Samples showing detectable growth were excluded from the study, as their results would reflect the activity of live cells rather than postbiotics.

### 2.4 Antimicrobial Test Method

The antimicrobial activity of postbiotic samples was evaluated using a modified Agar Diffusion Method, based on the protocol described by Gunes Altuntas et al. (2012) and Schillinger and Lücke (1987). In this approach, Soft‐Tryptic Soy Agar medium containing 0.1% indicator bacterial culture was uniformly spread over Tryptic Soy agar (TSA) plates. Wells with a diameter of 8 mm were created in the agar, and each was filled with 200 μL of the postbiotic preparation. The plates were then incubated at 30°C (for *L. monocytogenes* ) or 37°C (for other pathogen bacteria) for 24 h, depending on the specific pathogen being tested. Antimicrobial activity was assessed by measuring the diameter of inhibition zones formed around the wells.

### 2.5 Selection of Postbiotics Method

Postbiotic selection was based on cell viability and antimicrobial efficacy. Candidates showing viable cells were excluded, as food preservation depends on nonviable antimicrobial components. We then compared the antimicrobial performance of subgroups within each group, followed by a cross‐group comparison to identify the most effective formulations. This two‐step approach allowed us to determine the optimal preparation method for each bacterial strain.

### 2.6 Characterization of Postbiotics

The postbiotics in the form of CFS of the three bacteria were used for further characterization, including the determination of antioxidant activity, TPC, and the analysis of phenolic and flavonoid compounds for comparison purposes.

#### 2.6.1 Antioxidant Activity Assessment

The antioxidant activity of the postbiotic samples was evaluated using a modified DPPH (2,2‐diphenyl‐1‐picrylhydrazyl) radical scavenging method by Incili et al. (İncili et al. 2022). DPPH solution (2.5 mg/100 mL methanol) (Aldrich, Germany) was mixed with 100 μL of postbiotic samples and Trolox standards (Sigman Aldrich, Switzerland) (0.01–0.09 mg/mL), then vortexed and incubated in the dark for 45 min. Absorbance was measured at 515 nm using methanol as a reference, and antioxidant activity was expressed as mg Trolox equivalents per mL (mg TEAC/L).

#### 2.6.2 Total Phenolic Content

The TPC of lyophilized postbiotic samples was determined using a modified Folin–Ciocalteu method (Sornsenee et al. 2021). Samples were diluted to 50 mg/mL in distilled water, and a reaction mixture was prepared in a 96‐well microtiter plate with 100 μL of 0.1 M sodium carbonate and 100 μL of 10% Folin–Ciocalteu reagent (Sigma‐Aldrich, USA). Following a 1‐h incubation at room temperature, absorbance was recorded at 750 nm using a TECAN Infinite M Plex plate reader. A gallic acid calibration curve (2–200 μg/mL) was used to quantify TPC, expressed as gallic acid equivalents (GAE) per gram of lyophilized sample.

#### 2.6.3 Determination of the Phenolic and Flavonoid Compounds of Postbiotics

Fourteen phenolic and flavonoid compounds in the postbiotic samples were analyzed using two different methods. Chlorogenic acid, Caffeic acid, Vanillic acid, and Luteolin were examined through mass spectrometry (AGILENT App.Note, 5991‐7518 EN) using an Agilent 6460 Triple Quadrupole LC‐MS/MS system. This system featured an ESI+ Agilent Jet Stream ionization source and was connected to an Agilent 1200 Series high‐performance liquid chromatography (HPLC). Separation was achieved on a Zorbax SB‐C18 column (2.1 × 50 mm, 1.8 μm). The mobile phase consisted of Solvent A (0.05% formic acid + 5 mM ammonium formate) and Solvent B (methanol, MS grade). The column was maintained at a temperature of 40°C, with a flow rate of 0.5 mL/min using a gradient flow mode. The injection volume was 2 μL, and the total runtime for the analysis was 13 min. The mass spectrometry analysis was conducted in multiple reaction monitoring (MRM) mode, utilizing a gas temperature of 350°C, gas and sheath gas flow rates of 10 mL/min, a nebulizer pressure of 45 psi, a capillary voltage of 4000 V, and a nozzle voltage of 500 V. The standard curve range was from 0.002 to 1 ppm, and data acquisition and processing were performed using Agilent MassHunter Optimizer software. The phenolic and flavonoid compounds in the postbiotic sample were identified using the external standard method, with results expressed as ppm (Saini et al. 2023).

Additionally, organic acids—including acetic, butyric, formic, lactic, malic, oxalic, propionic, citric, succinic, and tartaric acids—were quantified using HPLC. The analysis was performed using an Agilent 1260 Infinity HPLC system equipped with a Metacarb 87H column (300 × 7.8 mm). The mobile phase consisted of 0.008N H_2_SO_4_, with a flow rate of 0.6 mL/min. The column temperature was maintained at 60°C, and detection was carried out at 210 nm using a diode array detector (DAD). The total runtime for the analysis was 25 min, and the results were expressed in mg/mL (Coelho et al. 2018).

### 2.7 Statistical Analyses

An independent *t*‐test was conducted to compare the antimicrobial activity between the heat treatment subgroups: MHT and CHT, as well as between the CFS Group and CHT. The antimicrobial activity of CFS, along with its antioxidant activity and TPC, was compared across different postbiotic preparation methods using ANOVA, followed by Tukey's post hoc test. All statistical analyses were performed using SPSS software, version 28.0 (IBM Corp., released in 2021). A *p*‐value of less than 0.05 was considered statistically significant, and all analyses were conducted in triplicate. The determination of the phenolic and flavonoid compounds in the postbiotics was carried out in two replicates.

## 3 Results

### 3.1 Cell Viability Test Results

The effects of various postbiotic preparation methods on bacterial cultures were assessed through cell viability tests. Postbiotics derived from CFS and subjected to thermal treatment consistently showed negative results across all tested conditions, indicating the absence of viable cell growth. In contrast, postbiotic samples prepared using enzymatic treatment demonstrated continued cell growth. This result suggests that the enzymatic method was insufficient to eliminate viable cells, rendering it unsuitable for effective postbiotic production.

### 3.2 Antimicrobial Test Results

The antimicrobial test was conducted on two groups: CFS and heat‐treated postbiotics. Within the heat‐treated group, a comparison was made between subgroups to identify the most effective subgroup.

#### 3.2.1 CFS Postbiotics

**FIGURE 1:**
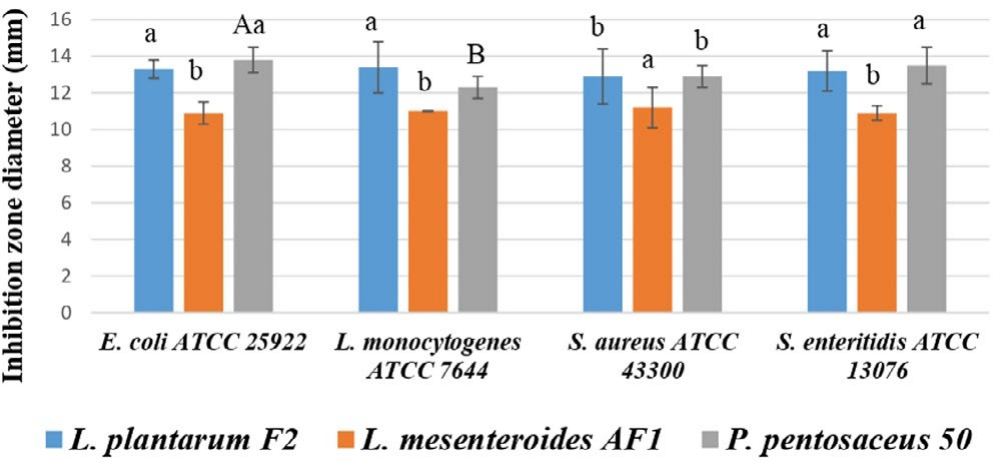
Antimicrobial test results of CFS, A–B: The mean values with different letters inside each CFS, a–b: The mean values with different letters among the CFS against pathogens are significantly different (*p* < 0.05).

The antimicrobial activity of CFS from different postbiotics was evaluated using a One‐Way ANOVA (*p* < 0.05), followed by Tukey's HSD post hoc analysis to identify significant differences between treatments (Figure 1). Notably, postbiotic *P. pentosaceus* 50 showed statistically significant differences in inhibition zones between *E. coli* ATCC 25922 and *L. monocytogenes* ATCC 7644 (13.8 ± 0.7 mm and 12.3 ± 0.6 mm, respectively; *p* < 0.05), highlighting its selective antimicrobial effect. When comparing the three CFS postbiotics across each pathogen, *L. plantarum* F2 and *P. pentosaceus* 50 exhibited significantly higher inhibition zones against *E. coli* ATCC 25922, *S. aureus* ATCC 43300, and *S. enteritidis* ATCC 13076 compared to *L. mesenteroides* AF1. For *L. monocytogenes* ATCC 7644, *L. plantarum* F2 demonstrated the highest inhibition zone, with a statistically significant difference from *L. mesenteroides* AF1. In the case of *S. aureus* ATCC 43300, *L. mesenteroides* AF1 showed a significantly lower inhibition zone compared to *L. plantarum* F2 and *P. pentosaceus* 50. This analysis underscores the distinct antimicrobial activities of the different postbiotics, with *P. pentosaceus* 50 exhibiting broad‐spectrum activity, particularly against *E. coli* and *S. enteritidis* ATCC 13076.

#### 3.2.2 Heat Treatment Method

**FIGURE 2:**
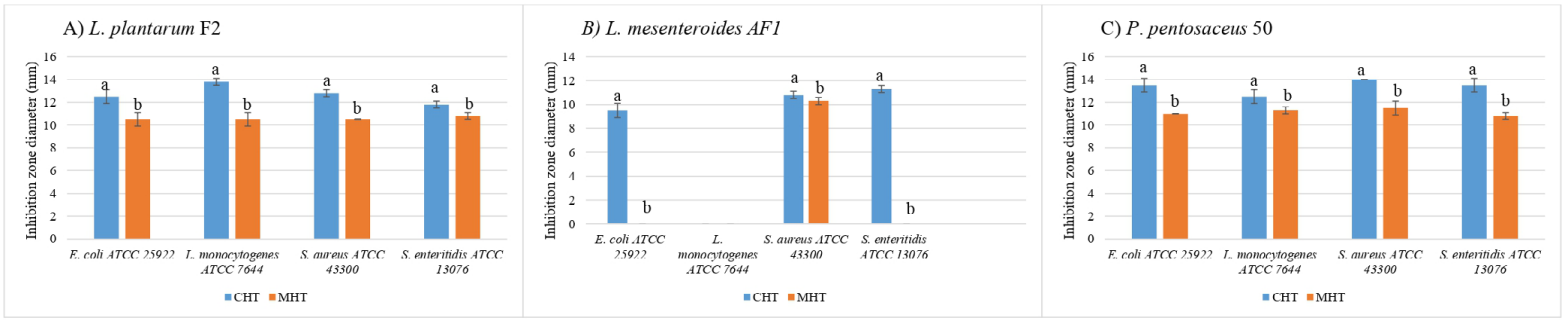
Antimicrobial test results of thermal treatment, a–b: The mean values with different letters inside each postbiotic are significantly different (*p* < 0.05). CHT, complete heating treatment; MHT, mixed heating treatment.

The heated cells subgroup of the heating treatment group showed no results in the antimicrobial test, so it was eliminated from Figure 2.

The antimicrobial activity of postbiotics subjected to CHT and MHT was compared for each LAB strain, with statistical analysis performed using the *t*‐test (*p* < 0.05) to identify significant differences (Figure 2).

For *L. plantarum* F2, CHT exhibited significantly higher inhibition against all tested pathogens compared to MHT (*p* < 0.05). In *L. mesenteroides* AF1, CHT showed statistically greater antimicrobial activity, particularly against *E. coli* (9.5 ± 0.6) and *S. enteritidis* (11.3 ± 0.3), where MHT had little to no effect (*p* < 0.05). For *P. pentosaceus* 50, CHT demonstrated significantly higher inhibition against all the pathogens (*p* < 0.05).

Overall, CHT was statistically more effective, consistently producing larger inhibition zones across the LAB strains and pathogens tested.

### 3.3 Selection of the Postbiotics Method

**FIGURE 3:**
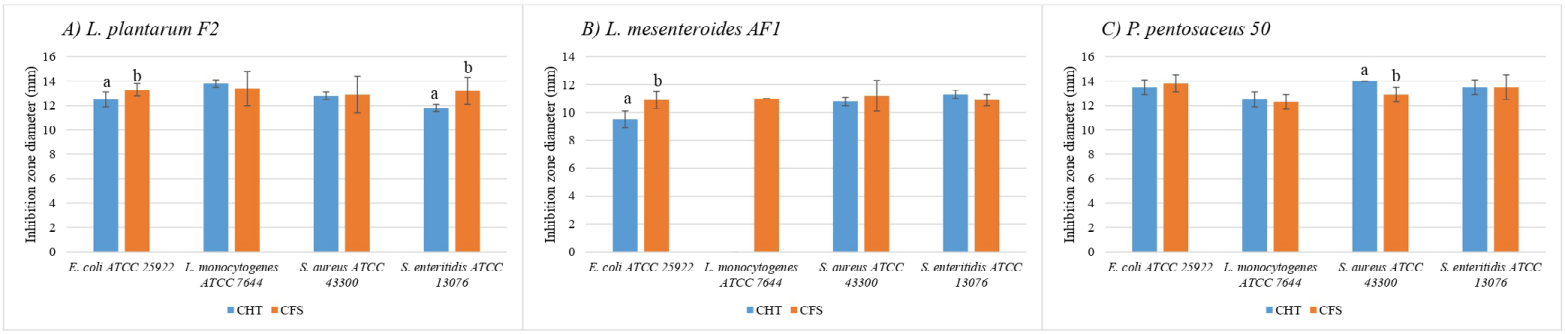
Antimicrobial test results of thermal treatment and CFS, a–b: The mean values with different letters inside each postbiotic are significantly different (*p* < 0.05). CFS, cell‐free supernatant; CHT, complete heating treatment.

All postbiotic candidates showing positive cell viability, specifically, the enzymatic treatment group and its subgroups, were excluded from further analysis. Among the remaining nonviable preparations, which included the heat‐treated subgroups and CFS, subgroup‐level comparisons revealed that within the heat‐treated group, the CHT subgroup demonstrated the highest antibacterial activity. Subsequently, cross‐group comparisons were conducted between the most effective heat‐treated subgroups, CHT and CFS, as shown in Figure 3.

The antimicrobial activity of postbiotics from *L. plantarum* F2, *L. mesenteroides* AF1, and *P. pentosaceus* 50 was compared between the CFS and CHT groups. Statistical analysis using the *t*‐test (*p* < 0.05) revealed significant differences in inhibition zones for several pathogens (Figure 3).

For *L. plantarum* F2, CFS showed significantly higher inhibition against *E. coli* ATCC 25922 (13.3 ± 0.5 mm) and *S. enteritidis* ATCC 13076 (13.2 ± 1.1 mm) compared to CHT (12.5 ± 0.6 mm and 11.8 ± 0.3 mm, respectively; *p* < 0.05). However, no significant difference was observed for *L. monocytogenes* ATCC 7644 or *S. aureus* ATCC 43300, with both treatments producing similar inhibition zones.

In the case of *L. mesenteroides* AF1, CFS demonstrated significantly higher activity against *E. coli* ATCC 25922 (10.9 ± 0.6 mm) compared to CHT (9.5 ± 0.6 mm, *p* < 0.05). Additionally, CFS showed inhibition against *L. monocytogenes* ATCC 7644 (11.0 ± 0.0 mm), while CHT had no effect. For *S. aureus* ATCC 43300 and *S. enteritidis* ATCC 13076, the differences between the two treatments were not statistically significant.

For *P. pentosaceus* 50, CHT showed slightly higher inhibition against *S. aureus* ATCC 43300 (14.0 ± 0.0 mm) compared to CFS (12.9 ± 0.6 mm, *p* < 0.05), indicating a better performance in this case. However, no statistically significant differences were observed for *E. coli* ATCC 25922, *L. monocytogenes* ATCC 7644, or *S. enteritidis* ATCC 13076, with both treatments showing comparable antimicrobial effects.

Overall, CFS generally exhibited greater antimicrobial activity, particularly against *E. coli* and *L. monocytogenes* in *L. mesenteroides* AF1 and *L. plantarum* F2, while CHT from *P. pentosaceus* 50 showed moderate activity against *S. aureus* .

### 3.4 Characterization of Postbiotics

#### 3.4.1 Antioxidant Activity Analysis Results

**FIGURE 4:**
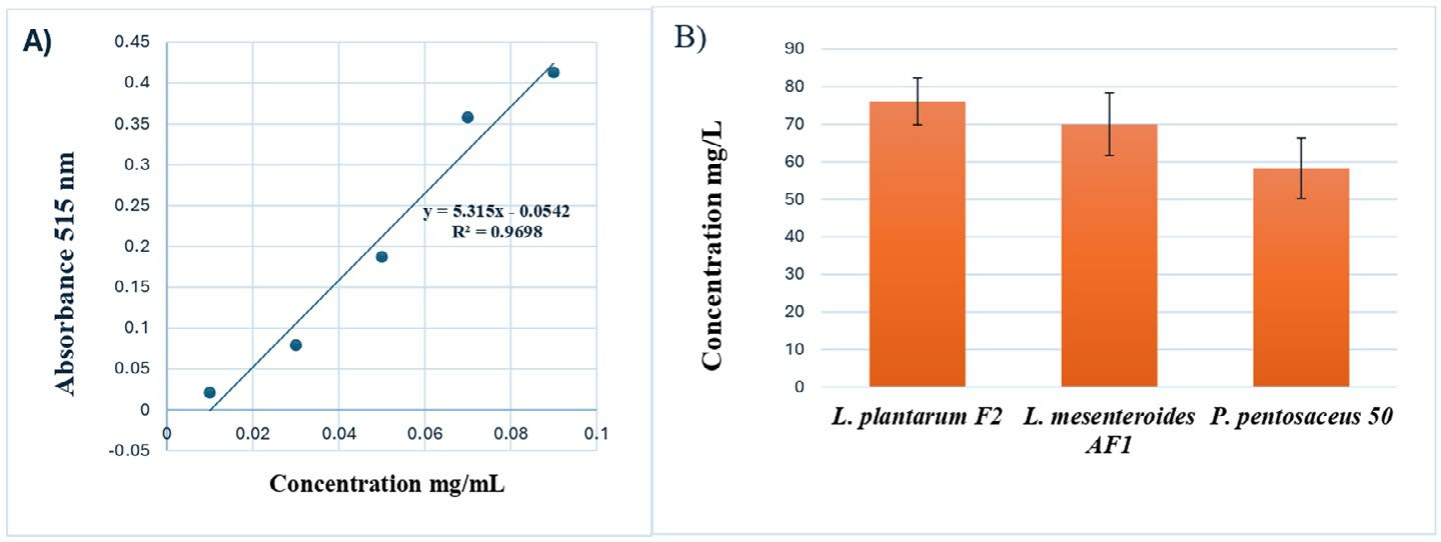
(A) The standard curve of DPPH used in the antioxidant activity assay. (B) Antioxidant results as mg TEAC/L (SD *n* = 3).

Antioxidant activity, measured as mg TEAC/L, was determined using a standard curve based on Trolox concentrations (0.01–0.09 mg/mL) (Figure 4A). One‐way ANOVA showed no statistically significant differences among the samples (*p* = 0.07). However, a trend toward higher antioxidant activity was observed in postbiotics derived from *L. plantarum* F2 isolates, which exhibited the highest antioxidant activity at 76.11 ± 6.21 mg/L, followed by *L. mesenteroides* AF1 at 70.03 ± 8.32 mg/L. *P. pentosaceus* 50 demonstrated the lowest antioxidant activity, measuring 58.30 ± 8.06 mg/L. These findings suggest that *L. plantarum* F2 may possess greater antioxidant potential compared to the other strains (Figure 4B).

#### 3.4.2 Total Phenolic Content

**FIGURE 5:**
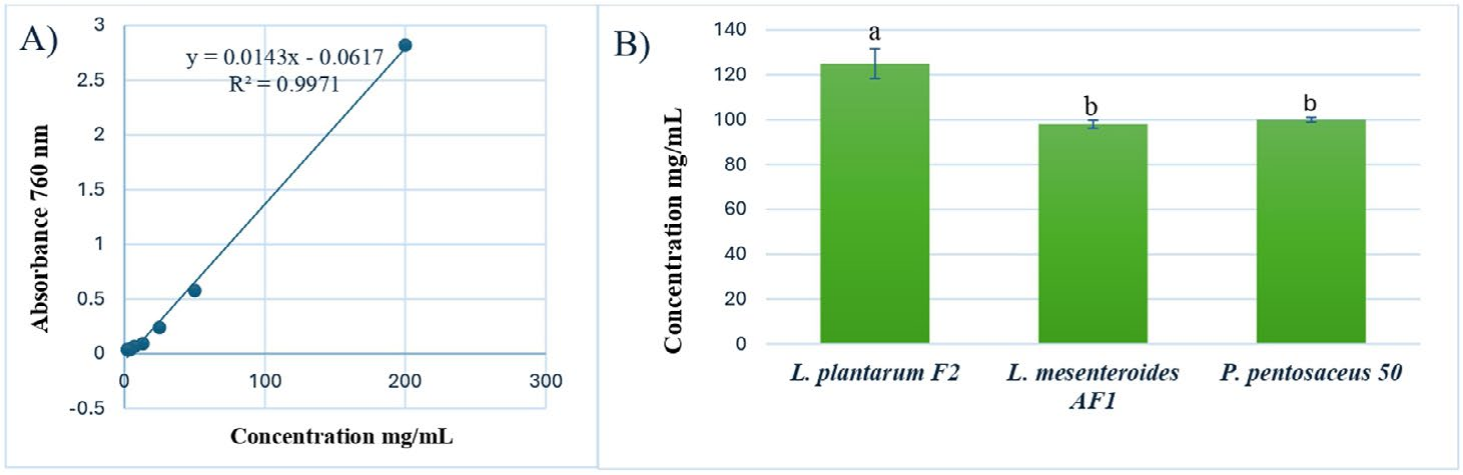
(A) The gallic acid standard calibration curve used in the total phenolic content assay. (B) Total phenolic constant results with SD *n* = 3 (Mean values with different superscripts (a–b) on the bars are significantly different at *p* < 0.001).

TPC values, expressed as GAE per gram of lyophilized sample, were calculated using a standard curve derived from gallic acid concentrations (2–200 μg/mL) (Figure 5A). One‐way ANOVA revealed significant differences among the three postbiotic samples (*p* < 0.001), with Tukey's HSD test indicating that *L. plantarum* F2 exhibited the highest TPC (125 ± 6.6 mg GAE/mL), which was significantly greater than that of *L. mesenteroides* AF1 (98 ± 1.8 mg GAE/mL) and *P. pentosaceus* 50 (100 ± 1.1 mg GAE/mL) (Figure 5B). These findings suggest that *L. plantarum* F2 postbiotics may possess superior phenolic content, potentially contributing to enhanced bioactivity.

#### 3.4.3 Determination of the Phenolic and Flavonoid Compounds of Postbiotics

A total of 14 different phenolic compounds were determined in Table 1. *L. plantarum* F2 exhibited the highest concentrations of acetic acid (6.25 ± 0.07 mg/mL) and lactic acid (17.2 ± 0.1 mg/mL), highlighting its superior production capacity for these organic acids compared to *L. mesenteroides* AF1 and *P. pentosaceus* 50. In contrast, *P. pentosaceus* 50 demonstrated the highest citric acid concentration (1.47 ± 0.04 mg/mL), closely followed by *L. mesenteroides* AF1 (1.44 ± 0.03 mg/mL), while *L. plantarum* F2 showed a comparatively lower level (0.86 ± 0.02 mg/mL).

**TABLE 1:** The phenolic and flavonoid contents of the postbiotics (mg/mL, ±SD [*n* = 2]).

Name	*L. plantarum* F2	*L. mesenteroides* AF1	*P. pentosaceus* 50
Chlorogenic acid	≤ 0.005	≤ 0.005	≤ 0.005
Caffeic acid	≤ 0.05	≤ 0.05	≤ 0.05
Vanillic acid	≤ 0.01	0.038 ± 0.000	0.054 ± 0.000
Luteolin	≤ 0.01	0.005 ± 0.001	0.010 ± 0.001
Acetic Acid	6.25 ± 0.07	6.00 ± 0.20	5.90 ± 0.20
Butyric Acid	N.D.	N.D.	N.D.
Formic Acid	N.D.	N.D.	N.D.
Lactic Acid	17.2 ± 0.1	11.2 ± 0.3	13.7 ± 0.42
Malic Acid	0.04 ± 0.01	N.D.	N.D.
Oxalic Acid	N.D.	N.D.	N.D.
Propionic Acid	N.D.	N.D.	N.D.
Citric Acid	0.86 ± 0.02	1.44 ± 0.03	1.47 ± 0.04
Succinic Acid	0.58 ± 0.05	1.10 ± 0.02	0.74 ± 0.08
Tartaric Acid	0.14 ± 0.01	0.13 ± 0.02	0.13 ± 0.01

Abbreviation: N.D., not detected.

The phenolic compound analysis revealed that chlorogenic acid and caffeic acid were present at concentrations below ≤ 0.005 mg/mL and ≤ 0.05 mg/mL, respectively, across all bacterial groups. Vanillic acid was detected only in *L. mesenteroides* AF1 and *P. pentosaceus* 50, with concentrations of 0.038 ± 0.000 mg/mL and 0.054 ± 0.000 mg/mL, respectively, while *L. plantarum* F2 had levels below the detection limit (≤ 0.01 mg/mL). Luteolin was quantified in *L. mesenteroides* AF1 (0.005 ± 0.001 mg/mL) and *P. pentosaceus* 50 (0.010 ± 0.001 mg/mL), whereas it remained below the detection threshold in *L. plantarum* F2 (≤ 0.01 mg/mL).

Organic acid profiling indicated notable differences among the bacterial groups. Acetic acid concentrations were slightly higher in *L. plantarum* F2 (6.25 ± 0.07 mg/mL) compared to *L. mesenteroides* AF1 (6.00 ± 0.20 mg/mL) and *P. pentosaceus* 50 (5.90 ± 0.20 mg/mL). Lactic acid production was most pronounced in *L. plantarum* F2 (17.2 ± 0.1 mg/mL), followed by *P. pentosaceus* 50 (13.7 ± 0.42 mg/mL) and *L. mesenteroides* AF1 (11.2 ± 0.3 mg/mL). Malic acid was detected exclusively in *L. plantarum* F2 (0.04 ± 0.01 mg/mL), while oxalic acid, propionic acid, butyric acid, and formic acid were not detected in any group.

Among other organic acids, citric acid levels were higher in *L. mesenteroides* AF1 (1.44 ± 0.03 mg/mL) and *P. pentosaceus* 50 (1.47 ± 0.04 mg/mL) compared to *L. plantarum* F2 (0.86 ± 0.02 mg/mL). Succinic acid was also elevated in *L. mesenteroides* AF1 (1.10 ± 0.02 mg/mL), followed by *P. pentosaceus* 50 (0.74 ± 0.08 mg/mL) and *L. plantarum* F2 (0.58 ± 0.05 mg/mL). Tartaric acid concentrations remained consistent across all groups, ranging from 0.13 ± 0.01 to 0.14 ± 0.01 mg/mL.

These findings demonstrate distinct metabolic profiles among *L. plantarum* F2, *L. mesenteroides* AF1, and *P. pentosaceus* 50, with notable differences in phenolic and organic acid production.

## 4 Discussion

The production of postbiotics requires the inactivation of probiotic cells while retaining their beneficial properties (Rafique et al. 2023). Among the commonly used methods, CFS and thermal treatment have proven to be the most effective, while enzymatic treatment combined with ultrasonication has shown limitations. Our findings confirm that CFS consistently produces postbiotics that are free of viable cells, making it a preferred method in line with previous research (Abbasi et al. 2023; Rocchetti et al. 2024). Thermal treatment has also been shown to effectively eliminate viable cells without compromising bioactive compounds when conducted at temperatures below 100°C, as reported by Sun et al. (2023). Almada et al. (2021) similarly found that heating *Lactobacillus acidophilus* , *Lacticaseibacillus casei*, and *Bifidobacterium animalis* at 80°C for more than 20 min effectively inhibited bacterial growth, while low‐intensity ultrasonication for short durations was insufficient. On the other hand, enzymatic treatment combined with ultrasonication exhibited limitations, likely influenced by factors such as enzyme type, duration, and ultrasonication intensity. Some studies applied enzymatic treatment without verifying cell viability (Aguilar‐Toalá et al. 2019), raising concerns about the source of the observed effects. Others combined filtration with enzymatic and ultrasonication treatments to obtain cell‐free extracts (Brandi et al. 2020; Li et al. 2012), highlighting that enzymatic treatment alone has lower efficacy. In conclusion, while CFS and thermal treatment are reliable methods for postbiotic production, enzymatic methods require refinement and further research to optimize protocols, ensuring the complete elimination of viable cells for safe and effective postbiotic applications.

The antimicrobial activity of postbiotics is influenced by both the preparation method and the bacterial strain used. In this study, a comparison between thermal treatment subgroups revealed that CHT postbiotics generally exhibited stronger inhibitory effects than MHT postbiotics. This trend suggests that the presence of heat‐treated cells without antimicrobial activity in MHT may dilute the bioactive compounds originally present in the CFS, thereby reducing its overall inhibition potential. In contrast, CHT appears to retain more of the antimicrobial efficacy, likely due to the preservation or activation of heat‐stable bioactive compounds.

When comparing CFS and CHT, variations in antimicrobial activity were observed depending on the bacterial strain and the target pathogen. CFS demonstrated greater antimicrobial efficacy against *E. coli* and *L. monocytogenes* in *L. mesenteroides* AF1 and *L. plantarum* F2, suggesting that bioactive compounds such as organic acids, bacteriocins, and other antimicrobial metabolites play a significant role in bacterial inhibition (Kim et al. 2020). In contrast, CHT exhibited a slight advantage in inhibiting *S. aureus* in *P. pentosaceus* 50, indicating that the antimicrobial compounds in this strain's supernatant might be more heat‐stable or even enhanced by heat treatment. This suggests the presence of thermostable bacteriocins or other heat‐resistant antimicrobial agents (Zaiton et al. 2011).

Interestingly, while our findings showed increased antimicrobial activity in CFS, Khakpour et al. (2024) reported the opposite, with heat‐treated *L. plantarum* postbiotics demonstrating greater efficacy than CFS. This discrepancy may be attributed to strain‐specific responses to heat treatment, which can influence both the release and stability of antimicrobial metabolites, even within the same bacterial species. Additionally, variations in antimicrobial effects among untreated bacterial strains have been reported, with differences depending on isolation sources, genetic diversity, metabolic activity, and environmental adaptation (Vasiee et al. 2018). These factors highlight the complex interactions between bacterial strains and their antimicrobial potential, reinforcing the idea that processing methods such as heat treatment can further modulate their efficacy in postbiotic formulations.

Our results align with previous studies, such as Vasiee et al. (2018), who investigated bacteria isolated from traditional Iranian fermented foods. For instance, the antimicrobial activity of *L. mesenteroides* A31 against *E. coli* and *S. aureus* showed similarities to our findings with *L. mesenteroides* AF1. Likewise, *L. plantarum* A41 and B25 exhibited comparable inhibition against *E. coli* , aligning with *L. plantarum* F2 in our study. However, *P. pentosaceus* A31 demonstrated lower antibacterial activity than *P. pentosaceus* 50 in our investigation. In contrast, *L. plantarum* TW29‐1, isolated from an Iranian traditional fermented product, exhibited higher antimicrobial activity against *E. coli* and *S. aureus* compared to *L. plantarum* F2 in our study (Saboktakin‐Rizi et al. 2021).

Further supporting our findings, a study on postbiotic application in chicken breast fillet preservation (İncili et al. 2023) reported similar trends for *P. pentosaceus* 50. In that study, CHT postbiotics from *P. acidilactici* (heated at 121°C) exhibited higher activity against *L. monocytogenes* ATCC 7644 compared to CFS, though the difference was not statistically significant. Similarly, for *S. aureus* ATCC 43300, CHT showed greater antimicrobial activity than CFS, aligning with our results, though our study demonstrated statistical significance. However, the study also found that CFS was more effective than CHT against *S. enteritidis* ATCC 13076, contradicting our findings, which showed similar inhibition zones for both treatments. These discrepancies may be attributed to differences in bacterial strains and their secreted bioactive compounds, emphasizing the importance of strain‐specific responses to heat treatment.

Overall, the observed variations in antibacterial activity may stem from strain‐dependent differences in antimicrobial compound production, fermentation conditions, and genetic factors influencing bacteriocin expression and other antimicrobial metabolites. These findings highlight the need for optimizing postbiotic preparation methods based on bacterial strain characteristics to maximize antimicrobial efficacy. Further research is required to identify key factors affecting postbiotic activity and to develop standardized preparation protocols for consistent and effective antimicrobial performance.

The antioxidant activity analysis revealed that *L. plantarum* F2 exhibited the highest activity (76.11 mg/L), followed by *L. mesenteroides* AF1 (70.03 mg/L), while *P. pentosaceus* 50 showed the lowest (58.30 mg/L), indicating strain‐dependent variation. Similar trends have been reported previously, with *L. plantarum* strains isolated from fermented dates (Arasu and Al‐Dhabi 2017) and sauerkraut (Zhou et al. 2023) demonstrating high antioxidant activities. *L. mesenteroides* has also exhibited strong antioxidant potential, as seen in *L. mesenteroides* MKSR from kimchi (Lee and Kim 2019). In contrast, *P. pentosaceus* strains have generally shown lower activity, consistent with the present findings (Yang et al. 2020). These observations reinforce the notion that *L. plantarum* and *L. mesenteroides* strains generally exhibit higher antioxidant capacity than *P. pentosaceus* , although variations may arise depending on strain‐specific metabolic characteristics.

The TPC analysis revealed a statistically significant difference among postbiotic samples (*p* < 0.004), with *L. mesenteroides* AF1 exhibiting the highest TPC (783.4 ± 28.6 mg GAE/L), followed by *L. plantarum* F2 (758.1 ± 31.1 mg GAE/L), both surpassing *P. pentosaceus* 50 (672.8 ± 5.1 mg GAE/L). These findings align with previous studies indicating strain‐specific variations in TPC production (Vijayalakshmi et al. 2023). However, differences have been reported in other fermentation substrates, where *P. pentosaceus* exhibited higher TPC than *L. mesenteroides* (De Montijo‐Prieto et al. 2023), highlighting the influence of fermentation conditions on phenolic compound synthesis.

For the phenolic and flavonoid compounds of postbiotics, *L. plantarum* F2 demonstrated superior production of lactic (17.2 ± 0.1 mg/mL) and acetic acids (6.25 ± 0.07 mg/mL) compared to *L. mesenteroides* AF1 and *P. pentosaceus* 50, indicating its strong metabolic activity. Similar trends have been observed in previous studies, suggesting that strain‐specific differences influence organic acid synthesis (Zhang et al. 2021). The absence of propionic and butyric acids in *L. plantarum* F2 contrasts with findings in *L. plantarum* strains, indicating unique metabolic pathways. Additionally, the presence of citric and succinic acids in *P. pentosaceus* 50 suggests broader metabolic diversity, emphasizing the importance of strain selection and fermentation optimization for enhancing postbiotic properties. Future studies are needed to further characterize the metabolic diversity of LAB in different environmental and industrial applications.

## 5 Conclusion

In conclusion, this study underscores the significant impact of preparation methods and bacterial strain selection on the postbiotic antimicrobial effect. CFS and thermal treatments emerged as the most reliable methods for producing postbiotics free of viable cells, while enzymatic treatment combined with ultrasonication demonstrated notable limitations, necessitating further refinement. The antimicrobial activity varied among strains, with CHT postbiotics generally exhibiting superior inhibitory effects, reaffirming the importance of strain‐specific responses to heat treatment. Antioxidant activity analysis revealed that *L. plantarum* F2 consistently exhibited the highest antioxidant capacity, lactic acid, and acetic acid production, highlighting its superior metabolic activity. Additionally, the significant differences observed in TPC across strains reinforce the influence of bacterial genetics and fermentation conditions. Collectively, these findings emphasize the necessity of tailoring postbiotic production methods to optimize bioactivity, ensuring maximum antimicrobial, antioxidant, and metabolic potential for diverse applications. Further research is imperative to standardize protocols and unlock the full therapeutic and industrial potential of postbiotics.

## Ethics Statement

This study does not involve any human or animal testing.

## Consent

Written informed consent was obtained from all study participants.

## Conflicts of Interest

The authors declare no conflicts of interest.

## Data Availability

All data generated and analyzed in this research are presented within this article. Additional data supporting the findings can be obtained from the corresponding author upon reasonable request.
